# Drug Values in the Treatment of Disease

**Published:** 1915-08-14

**Authors:** 


### The Hospital

The Workers' Newspaper of Administrative Medicine and Institutional Life, Administration, National Insurance and Health.

No. 1522, Vol. LVIII. SATURDAY, AUGUST 14, 1915. PRICE ONE PENNY

### DRUG VALUES IN THE TREATMENT OF DISEASE.

Concerning drugs and their remedial values there are among members of the medical profession many degrees alike of belief and of unbelief. On the one hand we find an unwavering and unhesitating confidence, and on the other a hard and relentless scepticism; and between these extremes all possible shades and gradations abound. These propositions are equally true both of drugs in general and of individual members of the group. Differences of opinion of this order, too, may be found even among those of the most exalted professional rank, and they display themselves in reference to questions which *a priori* would have been classed as elementary. Take a single example. Some few years ago there was arranged in one of our contemporaries a series of papers on the treatment of pneumonia, and among these was a contribution, written by a physician of eminence, in which the value of opium in the disease in question was strongly emphasised. But there was also a second article, written by a physician of equal eminence, and in this opium was presented as a drug having very dangerous qualities for the pneumonic patient. The contrast, it must be admitted, is bewildering; yet it could be matched by many similar parallels. It is therefore hardly surprising that practical medicine is apt at times to be regarded as an area within which there is no certain knowledge and where the use of remedies is very largely a matter of routine, of formality, and of personal prejudice or habit. So long as the discussion is conducted as an affair of words much might be said for this proposition, but when the matter is tested in the field of realities it assumes another and very different complexion. Doctrinal disputes may continue as questions of academic interest, but they pass into the background in the discussion of the individual clinical issue. The question here is a limited and practical one, and it is rare to find anything like a sharp difference of opinion in reference to the treatment which ought to be adopted. Nevertheless the circumstances which lead to conflicting therapeutic announcements are worthy of note, and the recognition of them on a more general scale might perhaps do something to mitigate their acuteness.

First has to be remembered the tendency of many of the more acute processes of disease to a spontaneous cessation or natural cure. In many instances this may occur, and as a matter of fact does occur, even though no remedial agents are prescribed. Hence it may readily happen that when in cases of this order remedies have been employed they receive a verdict far in excess of their deserts. What has been achieved by nature is placed to the credit of art. And if, in such circumstances, one practitioner has accustomed himself to prescribe in a certain fashion, while another has selected a different combination, each may easily become the advocate of a method which seems to be in opposition to that vaunted by his colleague. Quite possibly both are equally right and equally wrong—right in that both have done something towards a favourable issue and wrong in the degree of importance they attach to their respective prescriptions. Here is the opportunity for conflict, and the conflict is sometimes waged quite beyond the measure of its real importance. From such disputes the moral is sometimes drawn that medicinal treatment is a matter of no importance either one way or the other, and the sceptic finds another argument for his incredulity. But the conclusion is far from being justified. It may be quite true that no medicine can directly effect the essential pathological processes of, say, enteric fever or acute pneumonia, but it does not follow from this admission that drugs cannot help the patient towards a favourable issue. Confinement to bed will not in all probability shorten the course of either of these diseases, but he would be a foolhardy patient who rejected the proposal. Similarly, drugs, though not touching the central problem, may help to establish conditions which will favour a happy solution. They may secure rest, promote elimination, or relieve distress, and in these and similar ways may make no small contribution to the desired end. To discard and despise them is as unjustifiable as it is to exaggerate their powers, yet from the circumstances here described it is not difficult to see how differences relative to their values may readily arise. Exactly how great or how little has been their contribution it may be impossible to say with anything like scientific precision. It is sufficient that they help even in the most moderate degree, for at the bedside the problem is not the nice estimation of therapeutic values, but the promotion of the welfare of the individual patient by all measures which with any reasonable degree of probability can be regarded as likely to afford help towards this end.

Another reason for the appearance of doctrinal differences in questions of therapeutics is the impossibility of conducting a series of experiments for the purpose of establishing clean-cut and confident conclusions. To treat a first hundred cases of, say, pneumonia in one fashion, a second hundred in a different fashion, and so on, might appeal to the professional statistician, and does seem at first sight an appropriate means of arriving at certainty. Yet the proposal involves the large assumption that all patients suffering from pneumonia need one and the same method of treatment, which is exactly what medical experience denies. Hence, even were the experiment made, it would be a mere historical record without much illumination or practical guidance. Clearly, the experiment can never be made, for the duty of the practitioner is to help his patient and not to try to solve general therapeutic or pharmacological problems. He will therefore employ whatever seems advisable in the individual instance, for his concern is with the treatment not of diseases but of ailing and suffering men and women. On hearing all this the person in rude health may be inclined to repeat some of the well-worn pleasantries which are cultivated at the expense of medical practice, but will he maintain this facetious attitude when he is attacked with acute pneumonia? If not, he loses the opportunity to drive his arguments home.

